# Inpatient psychiatric bed capacity within CMS-certified U.S hospitals, 2011–2023: A cross-sectional study

**DOI:** 10.1371/journal.pmed.1004682

**Published:** 2025-07-23

**Authors:** Zoe Lindenfeld, Jonathan H. Cantor, Colleen M. McCullough, Jemar R. Bather, Ryan K. McBain

**Affiliations:** 1 Edward J. Bloustein School of Planning and Public Policy, Rutgers University, New Brunswick, New Jersey, United States of America; 2 RAND Corporation, Santa Monica, California, United States of America; 3 Center for Anti-racism, Social Justice & Public Health, New York University School of Global Public Health, New York, New York, United States of America; 4 Department of Biostatistics, New York University School of Global Public Health, New York, New York, United States of America; UNRWA (united nations relief and works agency for palestine refugees in the near east), JORDAN

## Abstract

**Background:**

Despite persistently high rates of mental illness and suicide, receipt of treatment for mental health conditions remains low. In this context, it is important to quantify the number of inpatient psychiatric beds (IPBs), and to understand differences in the number of IPBs throughout the U.S, as these provide critical evaluation, medication, and stabilization services.

**Methods and findings:**

This study used nationally-representative data drawn from the 2011−2023 Centers for Medicare and Medicaid Services’ Healthcare Cost Report Information System (HCRIS). From 2011−2023, while the total number of IPBs—in both psychiatric hospitals (PHs) and short-term acute care hospitals (STACHs)—did not change, the number IPBs within STACHs fell from 11.3 in 2011 to 9.06 in 2023. During this period, 846 counties (in which over 244 million individuals reside) experienced a decline in the rate of IPBs, while another 1,449 counties (in which 59 million individuals reside) never had IPBs. In regression models predicting the number of IPBs in STACHs and PHs, hospitals that received DSH payments (STACHs: IRR:1.93, 95% CI: 1.72, 2.15; PHs: IRR:1.40; 95% CI: 1.06, 1.84), had more full-time employees (STACHs: IRR:1.35, 95% CI: 1.31, 1.38; PHs: IRR:1.77; 95% CI: 1.75, 1.80) and were teaching STACHs (STACHs: IRR:1.78; 95% CI: 1.63, 1.95) had significantly more IPBs. In county-level regression models, counties with a lower percentage of Black residents (*β*: −21.15; 95% CI: −37.14, −5.16) had a significantly higher rate of IPBs. The absence of a causal design means we cannot assess the reasons behind changes in IPBs across time, and is a limitation of this study.

**Conclusions:**

This study provides an overview of the availability of IPBs throughout the U.S, as well as the number of individuals without access to IPBs. Findings indicate a dearth of STACH-based IPBs, particularly in areas with a greater proportion of racial minority residents.

## Author summary

**Why was this study done?:**

- For individuals with acute mental health needs, inpatient psychiatric services are an important component of the mental healthcare continuum.
- Poor access to inpatient psychiatric services has also been associated with higher rates of homelessness, suicide risk, and premature mortality at the population-level.
- However, there is a dearth of research measuring the presence of community-based inpatient psychiatric beds nationwide, as well as investigating hospital and county-level factors associated with greater availability.

**What did the researchers do and find?:**

- This study used nationally representative data drawn from the Centers for Medicare and Medicaid Services’ (CMS) Healthcare Cost Report Information System (HCRIS) to measure has the number and geographic availability of inpatient psychiatric beds within U.S. hospitals changed between 2011 and 2023; geographic differences in the availability of inpatient psychiatric beds within U.S. hospitals over time; and the relationship between inpatient psychiatric bed availability and hospital characteristics, county characteristics, and state policies related to the reimbursement of mental healthcare.
- We found that in 2023, there were 28.4 inpatient psychiatric beds per 100,000 persons in the U.S, and counties with a higher percentage of Black residents had significantly fewer inpatient psychiatric beds.
- Less than a quarter of all short-term acute care hospitals reported having any inpatient psychiatric beds, and hospitals with more inpatient psychiatric beds were significantly more likely to be teaching hospitals, receive DSH payments, and have a higher number of full-time employees.

**What do these findings mean?:**

- Our findings indicate that across the US there is a shortage of inpatient psychiatric beds, with only 28.4 beds per 100,000 population; this is more than 30 beds fewer than the optimal level of 60 beds per 100,000 that is supported in the literature.
- Given that inadequate inpatient psychiatric beds create additional challenges for people in need of mental health treatment, including longer emergency department wait times and higher rates of psychiatric readmissions, these results have important implications for individuals with mental illness across the US.
- Our findings should be interpreted alongside key study limitations; our measure of inpatient psychiatric beds does not reflect variation in local demand or the evolving role of outpatient and community-based alternatives to inpatient care, and we relied on self-reported administrative data from hospitals, which may be subject to reporting inaccuracies.

## Abstract 3

In a cross-sectional study, Zoe Lindenfeld and colleagues quantified the number of inpatient psychiatric beds in the USA between 2011 and 2023, and assessed spatial and temporal trends.

## Introduction

Mental illnesses, including any mental illness (AMI) and serious mental illness (SMI), are a significant and growing source of morbidity and mortality in the United States (U.S). According to the 2023 National Survey of Drug Use and Health, an estimated 22.8% of adults aged 18 and older had AMI, and 5.7% had an SMI [1]. The consequences of these conditions are severe: 21.9 million people experienced a major depressive episode in 2023, and 12.8 million had serious thoughts of suicide. Suicide in particular is a persistent public health problem, with deaths by suicide totaling 49,449 in 2022 [2]. Despite persistently high rates of mental illness and suicide, rates of treatment receipt for mental health conditions remain low. Specifically, 4.5 million Americans with SMI (28.9% of all adults with SMI) failed to receive mental health treatment in 2024 [1]. In this context, it is important to quantify the number of inpatient mental health beds, and to understand differences in the number of inpatient mental health beds throughout the U.S.

For individuals with acute mental health needs, inpatient psychiatric services–which are provided in specialized units within community hospitals as well as in psychiatric hospitals–are an important component of the mental healthcare continuum and provide critical evaluation, medication, and stabilization services [3]. Poor access to inpatient psychiatric services has also been associated with higher rates of homelessness, suicide risk, and premature mortality at the population-level [4]. A focus of previous research has been to measure the optimal number of inpatient psychiatric beds (IPBs) within a geographic area such as a county or state [5,6]. Estimates range, but in many settings, 60 psychiatric beds per 100,000 residents is considered optimal [7]. Yet, recent estimates indicate that the U.S. had approximately 22 psychiatric beds per 100,000 residents [8], and this may have declined as a result of inpatient psychiatric facility closures that occurred throughout the COVID-19 pandemic [9,10]. While studies have documented the closure of inpatient psychiatric facilities, they have mainly focused on state psychiatric hospitals, rather than including community hospitals that provide the majority of inpatient psychiatric care throughout the U.S [11]. Moreover, there is a dearth of research mapping the presence of community-based IPBs nationwide, as well as investigating hospital and county-level factors associated with greater availability.

In recent years, federal and state policies have targeted the accessibility of inpatient psychiatric services. At the federal level, the Patient Protection and Affordable Care Act (ACA) defines mental health treatment as an essential health benefit, and state-level Medicaid expansion ACA broadened coverage to low-income childless adults, a population with high levels of unmet need for mental health treatment. Since 2015, states have also been able to apply for Section 1115 waivers to use Medicaid funds for nonelderly residents receiving mental health treatment in institutions for mental diseases (IMDs), which was prohibited in the original Medicaid legislation [12]. As of 2024, waivers to pay for mental health treatment in IMDs are approved in 11 states and pending in six other states [13]. These policies may be important tools for advancing access to inpatient psychiatric services, and they are critical to consider in evaluating the availability of IPBs throughout the U.S.

In this study, we use comprehensive nationally representative data drawn from the Centers for Medicare and Medicaid Services’ (CMS) Healthcare Cost Report Information System (HCRIS) to answer several research questions. First, how has the number and geographic availability of IPBs within U.S. hospitals changed between 2011 and 2023? Second, what geographic differences exist in the availability of IPBs within U.S. hospitals, and how have they changed over time? Finally, what is the relationship between IPB availability and hospital characteristics, county characteristics, and state policies that are related to the reimbursement of mental healthcare (e.g., Medicaid expansion, and 1115 IMD waivers) [13–15], as these factors have been associated with higher capacity for other hospital-based behavioral health services (i.e., substance use disorder services, pediatric inpatient psychiatry) [16–18].

## Methods

### Data sources/measurement

We used data from CMS’s HCRIS, compiled and processed through RAND’s Hospital Data repository [19]. The RAND Hospital Data team compiles all available years of data for all hospitals that have filed cost reports in HCRIS from 1996 forward, and use weighted sums and means to standardize all reported values to the calendar year. Missing data are not imputed, and no additional data audits or validation checks are performed by the RAND Hospital Data Team [19], given CMS requires hospitals complete accurate and timely costs reports, and failure to do so can result in up to 100% recall of CMS funds [20]. We analyzed data from 2011–2023, a period during which CMS had consistent and specific reporting requirements for IPBs [21].

### Participants

The sample included all CMS-certified hospitals (i.e., are enrolled in Medicare and/or Medicaid) that completed at least one cost report between 2011 and 2023. We excluded Veterans Administration (VA), military and Indian Health Services (IHS) hospitals because they are not required to complete annual cost reports. We identified IPBs in STACHs and psychiatric hospitals using standardized CMS cost report fields, following federal reporting definitions set by CMS. Classification does not vary by state.

### Variables

We extracted yearly data on the following characteristics: hospital type (e.g., community hospital, psychiatric hospital, teaching hospital, critical access hospital); total number of full time employees, total beds (which included general medical/surgical and intensive care unit beds for adults or children, and did not include long-term care, inpatient rehabilitation, skilled nursing facility, or hospice beds), and inpatient psychiatric unit beds; whether a hospital received disproportionate share hospital (DSH) payments, financial assistance given to hospitals that treat a large number of low-income patients, including those who are uninsured or Medicaid enrollees (an indicator of safety-net providers) [22]; ownership (e.g., government-owned, non-profit, for-profit), whether the hospital had an inpatient psychiatric unit (if not categorized as a psychiatric hospital). We include ownership status as a covariate due to research document changes in the composition of psychiatric inpatient facilities; for-profit facilities have grown relative to nonprofit ones, and studies have demonstrated that these facilities have increased rates of Medicaid acceptance over time [13].

We included established county-level demographic and socioeconomic characteristics as covariates in our analysis, drawn from the American Community Survey 5-year estimates from 2011–2023. The measures included data on the percentage of county residents who were Black, uninsured, and reported an income below the federal poverty limit. We also included an indicator for whether a hospital was located in a rural county, drawn from the U.S Department of Agriculture Rural–Urban Continuum Codes [23]. We then included two state-level policy indicators making payment for inpatient psychiatric services more feasible: (1) state Medicaid expansion status [24], and (2) state adoption of an IMD mental health waiver in a given year. We sourced data on IMD waivers from KFF and state websites [25].

Lastly, we obtained data on deaths by suicide at the county level from the Centers for Disease Control and Prevention (CDC) Restricted Access Mortality Files [26]. We chose to use this data to have the broadest inclusion of counties, even those with fewer than 10 deaths, which would be suppressed in the publicly available data. CDC mortality data drawn from death certificates, which are collected and coded by states and then sent to the CDC. Each death certificate contains information on the underlying cause of death using ICD-10 codes, and for this analysis, we only included deaths that had an ICD-10 code indicating death by suicide: U03, X60–X84, and Y87.0 [27]. We aggregated deaths at the county level for each year of data, and converted this measure to a death rate per 100,000 population using county-level population estimates drawn from the American Community Survey.

### Statistical methods

We calculated the national number of IPBs per 100,000 population in the U.S. each year (2011–2023). We separately calculated the number of IPBs per 100,000 population within short-term acute care hospitals (STACHs) and psychiatric hospitals. To estimate the average annual percentage change in IPBs over time, we fit a Poisson regression model relating IPBs to time, and calculated the percent change using the formula (*expβ* − 1) × 100 [28]. Next we calculated the total number of IPBs per 100,000 population (and separately in both STACHs and psychiatric facilities) for each U.S. state, county, and Hospital Referral Region (HRR) over the same period [29]. We conducted descriptive statistics on the organizational and county-level characteristics for all STACHs, STACHs with IPBs, and psychiatric hospitals in our dataset. We conducted all analyses using complete case analysis; no imputation was performed for missing data.

We estimated two mixed-effects negative binomial regression models with year fixed effects and random intercepts for state to assess the associations of independent variables of interest (organizational characteristics, county characteristics, and state policies) with two outcomes: number of IPBs within STACHs, and number of IPBs within psychiatric hospitals. We included an indicator for hospital size (i.e., bed count) as a covariate in these models. In these models, we exponentiated the coefficients to aid interpretability; as such, the coefficients reported are incident rate ratios (IRRs). We also explored effect modification by testing the interaction between time and Medicaid expansion, and time and IMD waiver status.

We then estimated a linear mixed-effects model to understand trajectories of beds per 100,000 population within U.S. counties, with year fixed effects, random effects at the county and state levels, and counties nested within states [30]. In this model, we included county demographic variables as independent variables: an indicator for rurality, an indicator for year, and state level policies. We also explored effect modification by testing the interaction between time and policy indicators (Medicaid expansion, IMD waiver). We conducted this analysis at the county level for three reasons. First, counties are a policy-relevant unit in many states (e.g., California, Pennsylvania, Texas), where behavioral health systems are administered and funded at the county level [31]; as such, when public-sector beds are scarce, residency status can affect priority or eligibility. Second, counties provide a finer level of geographic resolution (>3,000 units) than other levels of geographical organization such as HRRs, allowing for more granular assessment of geographic variation, especially in rural areas. Third, key sociodemographic variables included in our model are consistently available at the county level.

Lastly, we fit a linear mixed-effects model with county-level deaths by suicide per 100,000 population as the outcome to examine the relationship between IPB availability and suicide rates. This model included year fixed effects, random effects at the county and state levels, and counties nested within states. We included county demographic variables as covariates, as well as an indicator for rurality. Analyses were conducted with Stata version 18.0; *P* < .05 defined statistical significance (2-sided). The study was deemed exempt from IRB review by Rutgers University. This study is reported as per STROBE guideline (S1 STROBE Checklist)

## Results

### IPBs in the U.S. over time, overall and by facility type

**Fig 1:**
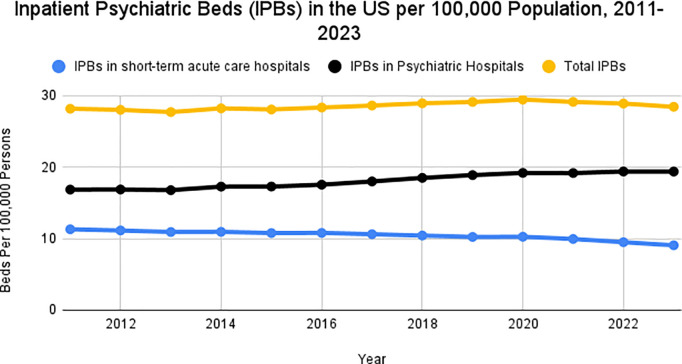
Inpatient Psychiatric Beds (IPBs) per 100,000 residents over time in US, 2011–2023. Average annual percent change for IPBs: IPBs in short-term acute care hospitals – −2.0 (95% CI: −5.84, 3.01); IPBs in psychiatric hospitals – 1.4 (95% CI: −1.9, 4.9); Total IPBs – 0.34 (95% CI: −2.34, 3.11).

Fig 1 documents the number of IPBs in psychiatric hospitals, IPB beds in STACHs, and total IPBs (within both psychiatric hospitals and STACHs) per 100,000 persons in the U.S. from 2011 to 2023. In 2011, there were 28.1 IPBs per 100,000 persons across both facility types, and in 2023 there were 28.4 IPBs per 100,000 persons across both facility types. The number of IPBs per 100,000 persons in psychiatric hospitals increased over time: from 16.8 in 2011 to 19.5 in 2023. By contrast, the number of STACH-based IPBs per 100,000 persons decreased over time, from 11.2 in 2011 to 8.9 in 2023. For all types of IPBs, the average annual percent change over the study period was not significant (Fig 1).

**Fig 2:**
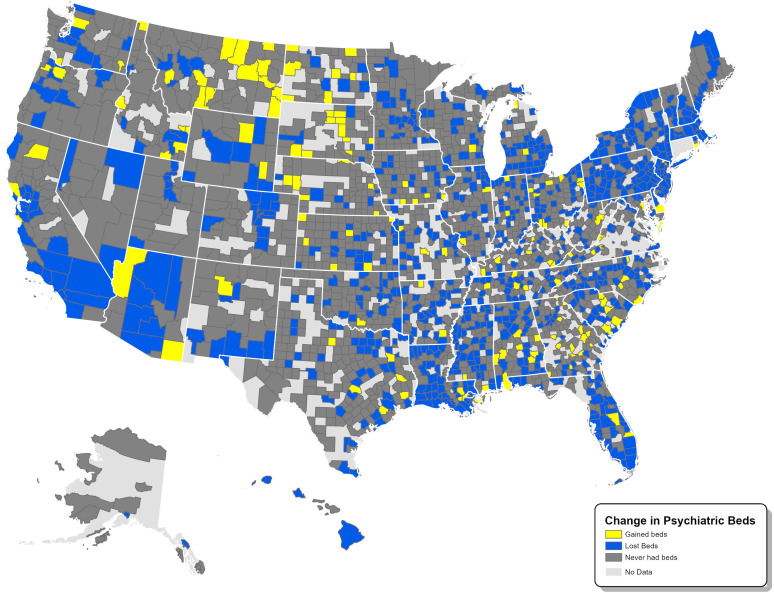
Change in inpatient psychiatric beds per 100,000 persons in U.S counties from 2011–2023. Map base layers: state – U.S. Census Bureau, “tl_2024_us_state,” TIGER/Line Shapefiles, 2024, https://www2.census.gov/geo/tiger/TIGER2024/STATE/, accessed on January 1, 2024.; county – U.S. Census Bureau, “tl_2024_us_county,” TIGER/Line Shapefiles, 2024, https://www2.census.gov/geo/tiger/TIGER2024/COUNTY/, accessed on January 1, 2024.

Fig 2 displays the change in total IPBs per 100,000 persons between 2011 and 2023 within U.S. counties; 846 counties (34.3%) experienced a decline in this rate, 165 (6.7%) experienced an increase in this rate, and 1,449 (58.9%) never had beds. Based on 2023 county population totals, this corresponds to 244,500,000 individuals who experienced a decline in IPBs, 13,969,821 individuals who experienced an increase in IPBs, and 59,469,960 who lacked access to IPBs entirely.

**Fig 3:**
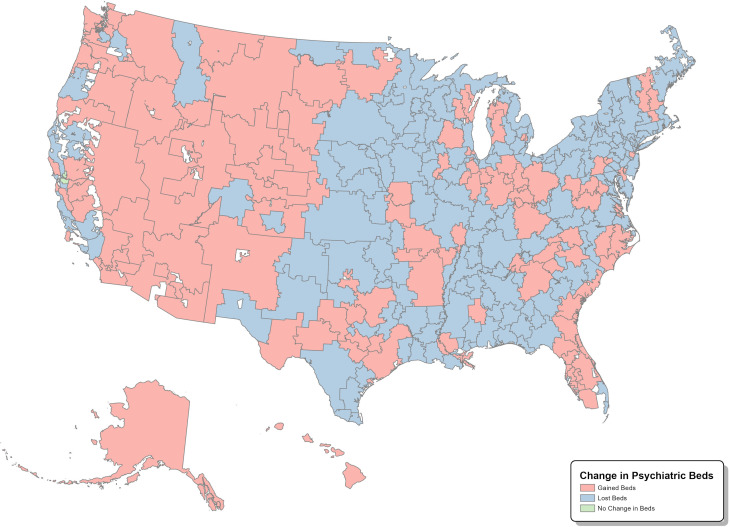
Change in total number of inpatient psychiatric beds in U.S Hospital Referral Regions (HRRs) from 2011 to 2023. Map base layer: https://data.dartmouthatlas.org/supplemental/.

Fig 3 displays the change in total IPBs between 2011 and 2023 within these regions. Across the study period, the mean number of IPBs in HRRs was 4,701.68 (SD: 99901.86); 40.20% of HRRs gained IPBs over the study period (*n* = 123), 59.48% (*n* = 182) lost IPBs, and 0.33% (*n* = 1) had no change in IPBs. S1 Table reports the total number of IPBs per 100,000 population in STACHs and psychiatric facilities for each U.S. state, as well as the percent change in the availability of IPBs between 2011 and 2023. Across years, 49.01% (*n* = 25) of states experienced a percentage decrease in total IPB per 100,000 population, 76.47% (*n* = 39) experienced a percentage decrease in the number of IPBs in STACHS, and 35.29% (*n* = 18) had a decrease in IPBs in psychiatric hospitals.

Descriptive statistics for hospitals included in our study are reported in Table 1. Our sample included 59,632 STACH-year observations, among which 13,878 (23.27%) reported having IPBs, and 7,044 psychiatric hospitals, all of which reported having IPBs. Compared to all STACHs, a higher percentage of STACHs with IPBs were teaching hospitals (49.50% versus 26.18%), had non-profit ownership (60.82% versus 48.50%), for-profit ownership (21.45% versus 19.47%), and received DSH payments (87.48% versus 59.50%); and a lower percentage of STACHs with IPBs were critical access hospitals (5.25% versus 28.50%) and had government ownership (17.23% versus 22.50%). STACHS with IPBs had a higher mean number of full-time employees (Mean: 1,877.46 [SD: 3,666.24] versus Mean: 976.29 [SD: 3,067.31]), and beds (Mean: 384.01 [SD:13,572.29] versus Mean: 176.41 [SD:6,553.39]). STACHs with IPBs had a mean of 31.35 IPB beds (SD: 32.92). Among psychiatric hospitals, 9.71% were teaching hospitals, 31.49% had government ownership, 14.01% had non-profit ownership, 54.50% had for-profit ownership, and 0.27% received DSH payments. Psychiatric hospitals had a mean number of 384.01 (SD: 13,572.29) beds, 1,223.53 (SD: 21919.37) full time employees, and 1,854.72 (SD: 2,081.92) yearly discharges. State-level policy and county-level demographic characteristics were similar across all three hospital types, though 40.25% of all STACHs were located in rural counties, compared with 24.6% of STACHs with IPUs and 12.4% of psychiatric hospitals.

**Table 1:** Descriptive statistics by hospital type, 2011–2023.

Variable	All short term acute care hospitals	Short term acute care hospitals with inpatient psychiatric beds	Psychiatric hospitals
***N***	59,632 (100%)	13,878 (23.27%)	7,044 (100%)
**Teaching hospital**	15,613 (26.18%)	6,856 (49.50%)	684 (9.71%)
**Critical access hospital**	16,796 (28.17%)	728 (5.25%)	0 (0%)
**Ownership**			
* Government owned*	13,387 (22.45%)	2,460 (17.73%)	2,218 (31.49%)
* Non-profit*	34,636 (48.08%)	8,441 (60.82%)	987 (14.01%)
* For-profit*	11,609 (19.47%)	2,977 (21.45%)	3,839 (54.50%)
**Receives DSH payments**	35,450 (59.45%)	12,141 (87.48%)	19 (0.27%)
**Full time employees** (Reports mean and standard deviation)	976.29 (SD: 3067.31)	1877.469 (SD: 3666.24)	1223.53 (SD: 21919.37)
**Total beds** (Reports mean and standard deviation)	176.41 (SD: 6553.39)	384.01 (SD: 13572.2)	108.38 (SD: 104.46)
**Inpatient psychiatric unit beds** (Reports mean and standard deviation)	—	31.35 (SD: 32.92)	—
**Rural**	24,002 (40.25%)	3,417 (24.62%)	877 (12.45%)
**% Uninsured in surrounding county**	11.52 (5.56%)	10.90 (5.18%)	11.06 (5.11%)
**% Households with income under FPL in surrounding county** (Reports mean and standard deviation)	11.02 (SD: 4.92)	11.35 (SD: 5.03)	10.64 (SD: 4.17)
**% Black in surrounding county** (Reports mean and standard deviation)	10.58 (SD: 13.71)	14.24 (SD: 14.95)	14.31 (SD: 13.79)
**% White in surrounding county** (Reports mean and standard deviation)	77.45 (SD: 17.46)	73.38 (SD: 17.50)	72.40 (SD: 15.99)
**% Hispanic in surrounding county** (Reports mean and standard deviation)	12.08 (SD: 19.47)	11.81 (SD: 13.56)	13.14 (SD:14.17)
**Located in state with medicaid expansion**	27,081 (SD: 45.41)	6,621 (SD: 47.71)	3,325 (SD: 50.04)
**Located in state with 1115 IMD waiver**	3,612 (6.06%)	575 (4.14%)	499 (7.08%)

### Modeling IPBs in STACHs

In the mixed-effect negative binomial regression model reporting exponentiated coefficients with IPBs in STACHs as the outcome, controlling for hospital size, STACHs that were teaching hospitals (1.78; 95% CI: 1.63, 1.95; *p* < 0.001), received DSH payments (IRR: 1.93; 95% CI: [1.72, 2.15]; *p* < 0.001), and had for-profit ownership compared to government ownership (IRR: 1.12; 95% CI: [1.01, 1.24]; *p* = 0.031) had significantly higher numbers of IPBs; STACHS with higher numbers of full-time employees (IRR: 1.35; 95% CI: [1.31, 1.38]; *p* < 0.001) also had significantly higher numbers of IPBs (Table 2). In contrast, STACHs that were critical access hospitals (IRR: 0.17; 95% CI: [0.15, 0.19]; *p* < 0.001), had non-profit ownership compared to government ownership (IRR: 0.83; 95% CI: [0.76, 0.91]; *p* < 0.001), and were located in rural counties (IRR: 0.72; 95% CI: [0.66, 0.79]; *p* < 0.001) had significantly fewer IPBs. S3 Table reports the results from models that include interaction terms between time and Medicaid expansion, and time and IMD waiver status; these interaction terms were not significant in any model.

**Table 2:** Mixed-effect negative binomial regression models predicting the number of psychiatric beds within short-term acute care hospitals and psychiatric hospitals, 2011–2023 (95% CI in parentheses).

Variable	Psychiatric beds within short term acute care hospitals	Beds within psychiatric hospitals
**Number of hospitals (*N*)**	54,676	6,457
**Teaching hospital**	1.78 (95% CI: [1.63, 1.95], *p* < 0.001)	1.08 (95% CI: [1.03, 1.14], *p* = 0.002)
**Critical access hospital**	0.17 (95% CI: [0.15, 0.19, *p* < 0.001)	—
**Ownership (based category: government owned)**		
* Non-profit*	0.83 (95% CI: [0.76, 0.91], *p* < 0.001)	0.54 (95% CI: [0.52, 0.57], *p* < 0.001)
* For-profit*	1.12 (95% CI: [1.02, 1.24], *p* = 0.031)	0.96 (95% CI: [0.93, 1.01])
**Receives DSH payments**	1.93 (95% CI: [1.72, 2.15], *p* < 0.001)	1.40 (95% CI: [1.06, 1.84], *p* = 0.16)
**Full time employees (log transformed)**	1.35 (95% CI: [1.31, 1.38], *p* < 0.001)	1.77 (95% CI: [1.75, 1.80], *p* < 0.001)
**Rural**	0.72 (95% CI: [0.66, 0.79], *p* < 0.001)	0.80 (95% CI: [0.76, 0.84], *p* < 0.001)
**% Uninsured in surrounding county**	0.99 (95% CI: [0.97, 1.00])	1.00 (95% CI: [0.99, 1.01])
**% Households with income under FPL in surrounding county**	1.01 (95% CI: [0.99, 1.01])	0.98 (95% CI: [0.97, 0.98], *p* < 0.001)
**% Black in surrounding county**	1.00 (95% CI: [0.99, 1.01])	1.00 (95% CI: [1.00, 1.01], *p* < 0.001)
**Located in state with Medicaid expansion**	1.03 (95% CI: [0.92, 1.15])	0.97 (95% CI: [0.93, 1.02])
**Located in state with 1115 IMD waiver**	0.94 (95% CI: [0.79, 1.13])	1.07 (95% CI: [0.99, 1.15])
**Year**	0.97 (95% CI: [0.96, 0.99], *p* < 0.001)	1.00 (95% CI: [0.99, 1.01])

VIFs for each model are reported in S2 Table.

Table reports Incident Rate Ratios.

Model predicting IPBs in STACHS: AIC – 176710.3; BIC – 176852.9.

Model predicting IPBs in psychiatric hospitals: AIC – 66424.89; BIC – 66526.49.

### Modeling IPBs in psychiatric hospitals

In the mixed-effect negative binomial regression model reporting exponentiated coefficients with IPBs in psychiatric hospitals as the outcome, hospitals that had nonprofit (IRR: 0.54; 95% CI: [0.52, 0.57]; *p* < 0.001) ownership compared to government ownership, and that were located in rural counties (IRR: 0.80; 95% CI: [0.76, 0.84]; *p* < 0.001) and counties with a higher percentage of households under the federal poverty limit (IRR: 0.98; 95% CI: [0.97, 0.98]; *p* < 0.001) had significantly fewer beds; and hospitals that were teaching hospitals (IRR: 1.08; 95% CI: [1.03, 1.14]; *p* = 0.002), received DSH payments (IRR: 1.40; 95% CI: [1.06, 1.84]; *p* = 0.016) and had higher numbers of full time employees (IRR: 1.77; 95% CI: [1.75, 1.80]; *p* < 0.001) had significantly higher numbers of beds.

### Modeling IPBs and deaths by suicide in U.S counties

In the longitudinal mixed-effects model estimating trajectories of total IPBs per 100,000 residents within U.S. counties (Table 3), counties with a higher percentage of Black residents (*β*: −21.15; 95% CI: [−37.14, −5.16]; *p* = 0.01) had significantly fewer IPBs per 100,000 population; counties with a higher percentage of uninsured residents (*β*: 51.26; 95% CI: [17.96, 84.56]; *p* = 0.003) had significantly higher IPBs per 100,000 population. Each year increase was associated with a significant decrease in psychiatric beds per 100,000 residents (*β*: −153.78; 95% CI: [−184.92, −122.65]; *p* < 0.001). S4 Table reports the results from models that include interaction terms between time and Medicaid expansion, and time and IMD waiver status.

**Table 3:** Mixed-effects model estimating trajectories of beds per 100,000 residents within U.S counties, 2011–2023*.

Variable	Coefficient (95% CI)
***N***	29,835
**% Black**	−21.16 (95% CI: [−37.14, −5.16], *p* = 0.01)
**% Uninsured**	51.26 (95% CI: [17.96, 84.56], *p* < 0.001)
**% Households with income under FPL**	−29.49 (95% CI: [−63.72, 4.72])
**Rural**	225.82 (95% CI: [−170.64, 622.28])
**Located in state with Medicaid expansion**	−161.22 (95% CI: [−425.50, 103.04])
**Located in state with 1115 IMD waiver**	−11.22 (95% CI: [−409.54, 387.09])
**Year**	−153.78 (95% CI: [−184.92, −122.65], *p* < 0.001)

* AIC – 613205.6; BIC – 613296.9.

VIFs reported in S2 Table.

In the mixed-effects model with county-level deaths by suicide per 100,000 population as the outcome, we did not find a significant association between the availability of IPBs in U.S. counties and deaths by suicide. These results are reported in S5 Table.

## Discussion

This study quantified trends in the number of IPBs in the U.S. over time, and identified hospital- and county-level characteristics associated with their availability. We found that as of 2023, there were 28.4 IPBs per 100,000 persons in the U.S. Compared to previous estimates this is over 30 fewer beds than what is considered optimal [7]. Additionally, 25 states experiencing a percentage decline in the total number of IPBs per 100,000 population over time. Furthermore, over the study period, 846 counties lost all IPBs, while 1,449 counties lacked IPBs entirely throughout the period, leaving 59,469,960 individuals without access. Given that inadequate IPBs create additional challenges for people in need of mental health treatment, including longer emergency department (ED) wait times, shorter hospital stays, and higher rates of psychiatric readmissions [4], these results have important implications for individuals with mental illness across the U.S.

Specifically, less than a quarter of all STACHS reported having any IPBs, which is troubling as the absence or shortage of hospital-based IPBs can contribute to patient boarding in emergency departments (EDs), which is expensive, does not lend itself to appropriate care, and limits available beds for people in need of other critical services [32,33]. However, the well-documented dearth and inaccessibility of community-based mental health treatment options leaves few options beyond the ED for patients in need of psychiatric treatment [33]. Indeed, mental health-related ED visits have increased over time in the US [34]. The IPB shortages identified in the current study present additional challenges for patients requiring psychiatric treatment, as EDs lack the therapeutic environment, activities, and specialized provider teams, including psychiatry consults, that are typically available in inpatient psychiatric units [35].

Notably, STACHS with more IPBs were more likely to be teaching hospitals, receive DSH payments, and have a higher number of full-time employees, indicating that issues related to capacity and funding may drive IPB shortages. Teaching hospitals have a multitude of purposes, which include educating and training future physicians, in addition to providing patient care. Given this dual mission, teaching hospitals typically have a more specialized workforce [36], and often partner with medical schools and other education programs to improve healthcare delivery. These additional resources, as well as training requirements for psychiatric residency programs, may enable teaching hospitals to justify retaining or increasing the number of psychiatric beds, particularly as inpatient psychiatric services offer lower reimbursement rates compared to medical or surgical services [37]. Similarly, receiving DSH payments may also better position hospitals to retain ICPs, as these funds can potentially help offset the financial loss of providing more psychiatric services, particularly as individuals with mental illness are disproportionately represented in Medicaid enrollment [38].

In contrast, we found that beds located in psychiatric hospitals increased over time, and that there were higher numbers of psychiatric hospital beds compared to IPBs in STACHS. Psychiatric hospitals are considered the ultimate safety-net for people with mental illness, offering services for patients whose needs exceed the capacity of community providers [8]. Patients are often referred to psychiatric hospitals via EDs for further evaluation, initiation or receipt of complex treatment, or diagnostic study [39]. Patients may also be remanded to state psychiatric hospitals by the state for involuntary civil commitment, or by the criminal justice system for pretrial competency services [39]. Given that psychiatric hospitals may serve multiple purposes, including both treatment and community safety [39], decreasing bed size may be harder to justify. Indeed, we found that government-owned psychiatric hospitals had significantly greater numbers of beds compared to non-profit or for-profit owned hospitals; this signals the potential importance of these facilities in the inpatient psychiatric treatment landscape, particularly in the absence of sufficient community-based services.

We also found important differences in the availability of beds based on county-level sociodemographic characteristics. In county-level models, we found that the number of beds per 100,000 population were fewer in counties with a greater percentage of Black residents. The dearth of psychiatric services within communities with a greater proportion of racial minority residents is well-established in the literature; for example, studies have found that counties with more Black and Hispanic residents have limited access to outpatient mental healthcare and substance use disorder treatment [17,40,41].

Our study both extends and contextualizes prior research. First, we document that the shortage of mental health services in counties with more racial minority residents also encompasses inpatient-psychiatric services. Second, given the established connection between poor access to mental healthcare and criminal justice involvement [42], our findings raise the question of whether limited availability of IPBs may contribute to this relationship in some communities. Future research should investigate this relationship further, and policymakers should evaluate opportunities to improve the mental health infrastructure in areas that lack these critical services. One way states tried to improve access to IPBs is Section 1115 waivers that enable states to use Medicaid funds to pay for inpatient mental health treatment in IMDs. However, within our models, hospitals located in states with these waivers did not have a statistically significantly greater number of beds. In part, this may be because the payments for psychiatric treatment within Medicaid are still too low for hospitals to justify increasing the availability of these services [43,44].

This analysis has several limitations that must be considered. First, cost reports are based on administrative records provided by hospitals, and as such, the data may be subject to human error and systematic inaccuracies; however, hospitals face substantial financial penalties for erroneous or delayed submissions, which mitigates this concern. Second, this hospital-level data does not include information on patient outcomes, and we could not measure how changes or differences in IPBs impact patient outcomes. The cost report also does not contain information about pricing for psychiatric services, which may impact the likelihood of their availability in a hospital. Additionally, because we do not include data from hospitals not required to complete an annual cost report (i.e., VA, military, and IHS hospitals), the number of IPBs in this study is an undercount. Third, while reporting definitions for IPF beds remained stable from 2011 to 2023, potential variation in reporting completeness over time may have introduced bias into the observed trends. Fourth, while our ideal measure of psychiatric bed access – 60 beds per 100,000 population—is supported in the literature [7], this measure does not reflect variation in local demand or the evolving role of outpatient and community-based alternatives to inpatient care. As such, demand for inpatient psychiatric services may vary substantially between counties and affect the appropriate number of psychiatric beds. Likewise, using counties to measure geographic access to IPBs introduces limitations, as counties may not capture patients’ typical travel patterns, and access may be influenced by resources in adjacent areas. Future work should incorporate local estimates of psychiatric service need and more comprehensive measures of access, including availability of outpatient and crisis services, to better assess alignment between capacity and demand. Additionally, we used county-level measures of racial composition as a proxy for disparities in access to psychiatric services, which may have introduced a risk of ecological fallacy. While these data provide insights into population-level patterns, future research should incorporate individual-level or stratified analyses to more directly assess racial disparities in access to psychiatric services. Finally, the absence of a causal design means the results presented are associations, and we cannot assess the reasons behind increases or decreases in IPBs across time. However, this presents an important avenue for future study.

Despite these limitations, this study is an important contribution to the literature in inpatient psychiatric services by documenting the decreasing availability of STACH-based IPBs within the U.S over time and the number of individuals without access to IPBs. It is particularly critical to understand the availability of these critical treatment resources in the context of anticipated cuts to federal and state behavioral health spending [45]. Our findings signal that additional effort is needed to increase access to these critical services, particularly in areas with a greater proportion of racial minority residents. In recent years, there has also been national interest in alternative solutions for behavioral health crises and mental illness, which include 988, community urgent walk-in centers, mobile crisis services, and crisis stabilization units [46]. Policymakers should consider these community-based services as reinforcements to inpatient psychiatric services, particularly in areas that otherwise lack stabilization and acute mental health services. Future research should connect these findings to patient outcomes, and incorporate a causal study design to examine the causal effect of key policy changes, such as Section 1115 IMD exclusion waivers, on the availability of IPBs in hospitals and counties nationwide.

## Supporting information

S1 Checklist STROBE Statement—Checklist of items that should be included in reports of cross-sectional studies.
(DOC)

S1 Table Mean number of inpatient psychiatric beds (within general hospitals) versus psychiatric hospital beds versus total beds (both inpatient psychiatric unit + psychiatric hospitals) (per 100,000 persons) in U.S states, 2011–2023. Standard deviations are reported in parentheses. Values represent the mean number of inpatient psychiatric beds per 100,000 population by state, calculated by first deriving population-weighted rates for each year and then averaging these values across all study years.
(DOCX)

S2 Table Variance inflation factors (VIF) for regression models. (DOCX)

S3 Table Mixed-effect negative binomial regression models predicting the number of psychiatric beds within short-term acute care hospitals and psychiatric hospitals with interaction terms between time and policy indicators, 2011–2023 (95% CI in parentheses). Table reports exponentiated coefficients. VIFs reported in S2 Table. Model 1: AIC – 177502.7; BIC – 177645.3. Model 2: AIC – 177503.5; BIC – 177646. Model 3: AIC – 66426.29; BIC – 66534.65. Model 4: AIC – 66426.66; BIC – 66535.02.
(DOCX)

S4 Table Mixed-effects model estimating trajectories of beds per 100,000 residents within U.S counties with interaction terms between time and policy indicators, 2011–2023. VIFs reported in S2 Table. Model 1: AIC – 613192.7; BIC – 613292.4. Model 2: AIC – 613198.8; BIC – 613298.4.
(DOCX)

S5 Table Mixed-effects model estimating suicide rates per 100,000 residents within U.S counties, 2011–2023. AIC – 598404.3; BIC – 598487.2. VIFs reported in S2 Table.
(DOCX)

## Abbreviations

ACA: Affordable Care Act
AMI: any mental illness
CDC: Centers for Disease Control and Prevention
CMS: Centers for Medicare and Medicaid Services
DSH: Disproportionate Share Hospital
ED: Emergency Department
HCRIS: Healthcare Cost Report Information System
HIS: Indian Health Services
HRR: Hospital Referral Region
IMD: Institutions for Mental Disease
IPB: inpatient psychiatric beds
PH: psychiatric hospitals
SMI: serious mental illness
STACH: short-term acute care hospitals
U.S: United States
VA: Veterans Administration
