# Supplementary material for: Inpatient psychiatric bed capacity within CMS-certified U.S hospitals, 2011–2023: A cross-sectional study
Source: PLoS Med. 2025 Jul 23;22(7):e1004682. doi: 10.1371/journal.pmed.1004682 (PMC12310024; doi:10.1371/journal.pmed.1004682)
Supplement: S1 Table — Standard deviations are reported in parentheses. Values represent the mean number of inpatient psychiatric beds per 100,000 population by state, calculated by first deriving population-weighted rates for each year and then averaging these values across all study years. (DOCX) [file pmed.1004682.s002.docx]

**S1 Table.** Mean number of inpatient psychiatric beds (within general hospitals) vs. psychiatric hospital beds vs. total beds (both inpatient psychiatric unit + psychiatric hospitals) (per 100,000 persons) in U.S states, 2011-2023

| **State** | **Inpatient Psychiatric Unit Beds** | **Percent Change from 2011-2023** | **Psychiatric Hospital Beds** | **Percent Change from 2011-2023** | **Inpatient Psychiatric Unit + Psychiatric Hospital Beds** | **Percent Change from 2011-2023** |
| --- | --- | --- | --- | --- | --- | --- |
| Alabama | 20.81 (SD: 0.54) | 7.30 | 11.89 (SD: 3.86) | -48.62 | 32.70 (SD: 3.47) | -19.02 |
| Alaska | 1.63 (SD: 0.01) | -1.50 | 22.47 (SD: 8.29) | 14.04 | 24.11 (SD: 8.30) | 13.11 |
| Arizona | 8.43 (SD: 0.94) | 14.08 | 19.05 (SD: 5.67) | 119.33 | 27.49 (SD: 6.49) | 79.40 |
| Arkansas | 16.80 (SD: 0.98) | 5.83 | 24.27 (SD: 2.43) | -1.15 | 41.08 (SD: 1.89) | 1.45 |
| California | 5.74 (SD: 0.19) | -10.34 | 9.35 (SD: 0.77) | 26.33 | 15.09 (SD: 0.66) | 10.85 |
| Colorado | 5.93 (SD: 0.40) | -2.48 | 19.54 (SD: 1.65) | 22.72 | 25.48 (SD: 1.93) | 16.14 |
| Connecticut | 21.81 (SD: 0.60) | -2.86 | 19.25 (SD: 3.32) | -31.09 | 41.06 (SD: 3.16) | -17.33 |
| Delaware | 2.72 (SD: 0.30) | 2.61 | 45.47 (SD: 5.88) | 19.29 | 48.19 (SD: 6.03) | 18.21 |
| Dist. of Columbia | 13.59 (SD: 5.03) | -51.59 | 43.79 (SD: 17.91) | 87.14 | 57.38 (SD: 18.56) | 32.46 |
| Florida | 11.08 (SD: 2.82) | -24.14 | 9.92 (SD: 1.25) | 39.29 | 21.01 (SD: 2.72) | 2.91 |
| Georgia | 6.93 (SD: 0.24) | -1.94 | 14.66 (SD: 0.65) | -3.12 | 21.60 (SD: 0.67) | -2.77 |
| Hawaii | 1.79 (SD: 0.37) | -35.91 | 6.20 (SD: 0.08) | -3.87 | 7.99 (SD: 0.42) | -12.01 |
| Idaho | 6.11 (SD: 0.45) | -8.29 | 18.51 (SD: 1.50) | 13.64 | 24.63 (SD: 1.73) | 7.48 |
| Illinois | 10.62 (SD: 0.89) | -13.44 | 14.89 (SD: 1.36) | 31.20 | 25.52 (SD: 1.44) | 10.49 |
| Indiana | 8.15 (SD: 1.44) | -45.66 | 21.09 (SD: 5.04) | 124.75 | 29.24 (SD: 3.70) | 54.09 |
| Iowa | 9.00 (SD: 1.94) | -52.07 | 6.51 (SD: 2.83) | -35.30 | 15.51 (SD: 4.09) | -44.53 |
| Kansas | 7.80 (SD: 1.39) | -39.58 | 11.15 (SD: 1.79) | -32.16 | 18.95 (SD: 2.89) | -35.28 |
| Kentucky | 9.02 (SD: 0.88) | -14.04 | 24.87 (SD: 1.46) | 13.54 | 33.89 (SD: 1.48) | 5.89 |
| Louisiana | 19.06 (SD: 0.68) | -3.02 | 44.92 (SD: 7.13) | 40.61 | 63.98 (SD: 7.06) | 26.33 |
| Maine | 5.97 (SD: 0.76) | 26.91 | 20.04 SD: (3.28) | -6.51 | 26.02 (SD: 3.64) | 0.12 |
| Maryland | 0.16 (SD: 0.41) | 0 | 29.14 (SD: 0.77) | -2.67 | 29.31 (SD: 0.86) | -2.67 |
| Massachusetts | 16.40 (SD: 0.89) | -16.19 | 24.81 (SD: 2.59) | 28.43 | 41.21 (SD: 2.07) | 8.66 |
| Michigan | 13.35 (SD: 0.86) | -21.14 | 17.59 (SD: 2.12) | 37.16 | 30.95 (SD: 1.39) | 8.73 |
| Minnesota | 10.03 (SD: 1.63) | -28.67 | 4.43 (SD: 0.56) | -31.98 | 14.47 (SD: 1.99) | -29.80 |
| Mississippi | 22.71 (SD: 2.57) | -33.36 | 17.20 (SD: 1.40) | 20.86 | 39.92 (SD: 1.58) | -12.29 |
| Missouri | 12.51 (SD: 2.61) | -45.73 | 21.04 (SD: 1.82) | -6.73 | 33.56 (SD: 3.91) | -22.18 |
| Montana | 4.68 (SD: 3.05) | -70.06 | 16.58 (SD: 0.61) | -11.16 | 21.66 (SD: 3.41) | -26.29 |
| Nebraska | 8.67 (SD: 0.63) | -12.95 | 11.17 (SD: 4.64) | 6.80 | 19.84 (SD: 4.94) | -1.34 |
| Nevada | 6.52 (SD: 0.82) | 46.88 | 30.91 (SD: 3.47) | 20.95 | 37.44 (SD: 3.90) | 25.01 |
| New Hampshire | 18.84 (SD: 12.86) | 10.84 | 16.14 (SD: 4.51) | 58.61 | 34.99 (SD: 15.67) | 33.34 |
| New Jersey | 13.21 (SD: 0.33) | 1.06 | 28.84 SD: (2.00) | -17.92 | 42.05 (SD: 2.11) | -12.34 |
| New Mexico | 9.76 (SD: 1.21) | -28.69 | 14.13 (SD: 0.85) | 19.28 | 23.89 (SD: 0.93) | -2.79 |
| New York | 24.64 (SD: 1.83) | -21.35 | 22.52 (SD: 1.36) | -16.26 | 47.16 (SD: 2.85) | -18.93 |
| North Carolina | 10.81 (SD: 0.69) | -18.34 | 17.80 (SD: 0.79) | 4.18 | 28.62 (SD: 0.58) | -4.43 |
| North Dakota | 14.61 (SD: 2.79) | -39.89 | 35.23 (SD: 4.74) | -30.23 | 49.85 (SD: 6.86) | -33.39 |
| Ohio | 11.76 (SD: 1.01) | -22.26 | 19.31 (SD: 3.92) | 78.40 | 31.07 (SD: 3.20) | 29.31 |
| Oklahoma | 9.32 (SD: 2.04) | -38.83 | 16.17 (SD: 2.54) | 27.83 | 25.50 (SD: 2.02) | -2.33 |
| Oregon | 7.41 (SD: 0.96) | 24.87 | 11.34 (SD: 5.33) | 281.12 | 18.76 (SD: 6.26) | 125.15 |
| Pennsylvania | 15.09 (SD: 1.45) | -29.72 | 27.76 (SD: 1.26) | -5.71 | 42.85 (SD: 1.70) | -14.26 |
| Rhode Island | 18.81 (SD: 3.03) | 18.89 | 13.34 (SD: 0.23) | 0.36 | 32.16 (SD: 3.06) | 10.30 |
| South Carolina | 10.07 (SD: 0.87) | -25.89 | 21.01 (SD: 1.83) | 21.58 | 31.09 (SD: 1.39) | 2.35 |
| South Dakota | 5.39 (SD: 2.41) | -56.64 | 10.09 (SD: 1.46) | -37.82 | 15.49 (SD: 3.75) | -45.11 |
| Tennessee | 14.09 (SD: 0.82) | -10.34 | 19.65 (SD: 2.60) | 13.95 | 33.74 (SD: 1.99) | 3.82 |
| Texas | 13.51 (SD: 18.09) | -93.34 | 20.53 (SD: 2.65) | 43.95 | 34.05 (SD: 15.93) | -62.96 |
| Utah | 9.55 (SD: 1.66) | 36.78 | 22.80 (SD: 3.22) | -9.84 | 32.36 (SD: 2.19) | 3.54 |
| Vermont | 3.63 (SD: 2.56) | -77.47 | 21.15 (SD: 2.94) | 57.27 | 24.79 (SD: 2.50) | 12.00 |
| Virginia | 10.70 (SD: 0.83) | -9.87 | 9.22 (SD: 1.04) | 22.90 | 19.93 (SD: 1.10) | 3.34 |
| Washington | 3.86 (SD: 0.23) | -9.28 | 18.03 (SD: 2.66) | 0.57 | 21.90 (SD: 2.69) | -1.45 |
| West Virginia | 20.79 (SD: 3.99) | -43.43 | 24.77 (SD: 4.81) | 38.48 | 45.57 (SD: 2.97) | -3.7 |
| Wisconsin | 2.98 (SD: 0.34) | -38.11 | 23.09 (SD: 0.97) | 2.09 | 26.08 (SD: 0.94) | -3.60 |
| Wyoming | 7.16 (SD: 1.40) | -33.84 | 26.72 (SD: 0.85) | 1.05 | 33.88 (SD: 1.51) | -7.39 |

**Legend:**

Standard deviation in parentheses

Values represent the mean number of inpatient psychiatric beds per 100,000 population by state, calculated by first deriving population-weighted rates for each year and then averaging these values across all study years.
