# Supplementary material for: Inpatient psychiatric bed capacity within CMS-certified U.S hospitals, 2011–2023: A cross-sectional study
Source: PLoS Med. 2025 Jul 23;22(7):e1004682. doi: 10.1371/journal.pmed.1004682 (PMC12310024; doi:10.1371/journal.pmed.1004682)
Supplement: S2 Table — (DOCX) [file pmed.1004682.s003.docx]

**S2 Table.** Variance inflation factors (VIF) for regression models

| **Variable** | **VIFs Model 1 (IPBs in STACHs)** | **VIFs Model 2 (IPBs in psychiatric hospitals)** | **VIFs Model 3 (IPBs in US Counties)** | **VIFs Model 4 (Suicide Rates in US Counties)-** |
| --- | --- | --- | --- | --- |
| Teaching hospital | 1.67 | 1.67 | — | — |
| Critical access hospital | 2.17 | — | — | — |
| Ownership  *Non-profit*  *For-profit* | 1.86  2.18 | 1.86  1.99 | — | — |
| Receives DSH Payments | 1.99 | 1.61 | — | — |
| Full time employees | 2.43 | 2.39 | — | — |
| Rural | 1.72 | 1.49 |  | 1.13 |
| % Uninsured in surrounding county | 2.11 | 2.10 |  | 1.60 |
| % Households with income under FPL | 2.05 | 2.05 |  | 1.90 |
| % Black in surrounding county | 1.58 | 1.55 |  | 1.44 |
| Located in state with Medicaid expansion | 1.58 | 1.58 |  | — |
| Located in state with 1115 IMD waiver | 1.08 | 1.08 |  | — |
| Year | 1.32 | 1.32 |  | 1.19 |
