# Supplementary material for: Inpatient psychiatric bed capacity within CMS-certified U.S hospitals, 2011–2023: A cross-sectional study
Source: PLoS Med. 2025 Jul 23;22(7):e1004682. doi: 10.1371/journal.pmed.1004682 (PMC12310024; doi:10.1371/journal.pmed.1004682)
Supplement: S3 Table — Table reports exponentiated coefficients. VIFs reported in S2 Table. Model 1: AIC – 177502.7; BIC – 177645.3. Model 2: AIC – 177503.5; BIC – 177646. Model 3: AIC – 66426.29; BIC – 66534.65. Model 4: AIC – 66426.66; BIC – 66535.02. (DOCX) [file pmed.1004682.s004.docx]

**S3 Table.** Mixed-effect negative binomial regression models predicting the number of psychiatric beds within short-term acute care hospitals and psychiatric hospitals with interaction terms between time and policy indicators, 2011-2023 (95% CI in parentheses)

| **Variable** | **Outcome: IPBs in STACHs** | **Outcome: IPBs in STACHs** | **Outcome : IPBs in Psychiatric Hospitals** | **Outcome: IPBs in Psychiatric Hospitals** |
| --- | --- | --- | --- | --- |
| **Teaching Hospital** | 1.86 | 1.86 | 0.08 | 0.08 |
|  | [95% CI: 1.71, 2.03]; p<0.001 | [95% CI: 1.71, 2.03]; p<0.001 | [95% CI: 0.03, 0.13]; p<0.001 | [95% CI: 0.03, 0.13]; p<0.001 |
| **Ownership (base category: government owned)** | | | | |
| *Non-profit* | 0.94 | 0.94 | -0.6 | -0.6 |
|  | [95% CI: 0.86, 1.02] | [95% CI: 0.86, 1.02] | [95% CI: -0.65, -0.56]; p<0.001 | [95% CI: -0.65, -0.56]; p<0.001 |
| *For-profit* | 1.45 | 1.45 | -0.03 | -0.03 |
|  | [95% CI: 1.31, 1.61]; p<0.001 | [95% CI: 1.31, 1.61]; p<0.001 | [95% CI: -0.07, 0.01] | [95% CI: -0.07, 0.01] |
| **Receives DSH payments** | 4.48 | 4.47 | 0.34 | 0.34 |
|  | [95% CI: 4.13, 4.85]; p<0.001 | [95% CI: 4.12, 4.85]; p<0.001 | [95% CI: 0.06, 0.61]; p=0.016 | [95% CI: 0.07, 0.61]; p=0.015 |
| **Full time employees (log transformed)** | 1.31 | 1.31 | 0.58 | 0.58 |
|  | [95% CI: 1.27, 1.34]; p<0.001 | [95% CI: 1.27, 1.34]; p<0.001 | [95% CI: 0.56, 0.59]; p<0.001 | [95% CI: 0.56, 0.59]; p<0.001 |
| **Rural** | 0.53 | 0.53 | -0.22 | -0.22 |
|  | [95% CI: 0.48, 0.58]; p<0.001 | [95% CI: 0.48, 0.58]; p<0.001 | [95% CI: -0.27, -0.17]; p<0.001 | [95% CI: -0.27, -0.17]; p<0.001 |
| **% Uninsured in surrounding county** | 0.98 | 0.98 | 0 | 0 |
|  | [95% CI: 0.97, 0.99]; p<0.001 | [95% CI: 0.97, 0.99]; p<0.001 | [95% CI: -0.00, 0.01] | [95% CI: -0.00, 0.01] |
| **% Households with income under FPL in surrounding county** | 1 | 1 | -0.02 | -0.02 |
|  | [95% CI: 0.99, 1.01] | [95% CI: 0.99, 1.01] | [95% CI: -0.02, -0.01]; p<0.001 | [95% CI: -0.02, -0.01]; p<0.001 |
| **% Black in surrounding county** | 1.01 | 1.01 | 0 | 0 |
|  | [95% CI: 1.01, 1.01]; p<0.001 | [95% CI: 1.01, 1.01]; p<0.001 | [95% CI: 0.00, 0.01]; p<0.001 | [95% CI: 0.00, 0.01]; p<0.001 |
| **Located in state with Medicaid expansion** | 0.93 | 0.01 | -0.02 | 4.43 |
|  | [95% CI: 0.83, 1.04] | [95% CI: 0.00, 2.5e+16] | [95% CI: -0.07, 0.02] | [95% CI: -13.56, 22.42] |
| **Located in state with 1115 IMD waiver** | 0 | 1.01 | -17.41 | 0.07 |
|  | [95% CI: 0.00, 2.9e+35] | [95% CI: 0.84, 1.22] | [95% CI: -61.32, 26.50] | [95% CI: -0.00, 0.15] |
| **Year** | 0.97 | 0.97 | 0 | 0 |
|  | [95% CI: 0.95, 0.98]; p<0.001 | [95% CI: 0.95, 0.98]; p<0.001 | [95% CI: -0.00, 0.01] | [95% CI: -0.00, 0.01] |
| **Interaction: Medicaid Expansion # Year** | | | | |
|  | --- | 1 | --- | 0 |
|  | --- | [95% CI: 0.98, 1.02] | --- | [95% CI: -0.01, 0.01] |
| **Interaction: IMD waiver # Year** | | | | |
|  | 1.03 | --- | 0.01 | --- |
|  | [95% CI: 0.96, 1.11] | --- | [95% CI: -0.01, 0.03] | --- |

**Legend:**

Table reports exponentiated coefficients

VIFs reported in Appendix Table 2.

Model 1- AIC- 177502.7; BIC- 177645.3

Model 2- AIC- 177503.5; BIC- 177646

Model 3- AIC- 66426.29; BIC- 66534.65

Model 4- AIC- 66426.66; BIC- 66535.02
