# Supplementary material for: Inpatient psychiatric bed capacity within CMS-certified U.S hospitals, 2011–2023: A cross-sectional study
Source: PLoS Med. 2025 Jul 23;22(7):e1004682. doi: 10.1371/journal.pmed.1004682 (PMC12310024; doi:10.1371/journal.pmed.1004682)
Supplement: S4 Table — VIFs reported in S2 Table. Model 1: AIC – 613192.7; BIC – 613292.4. Model 2: AIC – 613198.8; BIC – 613298.4. (DOCX) [file pmed.1004682.s005.docx]

**S4 Table.** Mixed-effects model estimating trajectories of beds per 100,000 residents within U.S counties with interaction terms between time and policy indicators, 2011-2023

| **Variable** | **Outcome: IPBs Per Capita in US Counties** | **Outcome: IPBs Per Capita in US Counties** |
| --- | --- | --- |
| **% Black** | -20.80 | -21.24 |
|  | [95% CI: -36.78, -4.82]; p=0.01 | [95% CI: -37.25, -5.23]; p=0.009 |
| **% Uninsured** | 47.41 | 51.31 |
|  | [95% CI: 13.93, 80.89]; p=0.006 | [95% CI: 18.00, 84.63]; p=0.003 |
| **% Households with income under FPL** | -29.14 | -29.52 |
|  | [95% CI: -63.37, 5.08] | [95% CI: -63.75, 4.71] |
| **Rural** | 226.78 | 225.42 |
|  | [95% CI: -169.62,623.18] | [95% CI: -171.11,621.96] |
| **Located in state with Medicaid expansion** | -160.66 | 33041.07 |
|  | [95% CI: -424.88,103.55]; | [95% CI: -7.1e+04, 1.4e+05] |
| **Located in state with 1115 IMD waiver** |  |  |
|  | -301439.4 | 16.07 |
|  | [95% CI: -580312.4,-22566.41]; p=0.03 | [95% CI: -391.25,423.40] |
| **Year** | -159.96 | -147.55 |
|  | [95% CI: -191.60,-128.31]; p<0.001 | [95% CI: -184.16,-110.94]; p<0.001 |
| **Interaction: Medicaid Expansion # Year** | --- | -16.46 |
|  | --- | [95% CI: -67.86,34.94] |
| **Interaction: IMD waiver # Year** |  |  |
|  | 149.16 | --- |
|  | [95% CI: 11.16,287.15]; p=0.03 | --- |

**Legend:**

VIFs reported in Appendix Table 2.

Model 1: AIC- 613192.7; BIC- 613292.4

Model 2: AIC- 613198.8; BIC- 613298.4
