# Supplementary material for: Inpatient psychiatric bed capacity within CMS-certified U.S hospitals, 2011–2023: A cross-sectional study
Source: PLoS Med. 2025 Jul 23;22(7):e1004682. doi: 10.1371/journal.pmed.1004682 (PMC12310024; doi:10.1371/journal.pmed.1004682)
Supplement: S5 Table — AIC – 598404.3; BIC – 598487.2. VIFs reported in S2 Table. (DOCX) [file pmed.1004682.s006.docx]

**S5 Table.** Mixed-effects model estimating suicide rates per 100,000 residents within U.S counties, 2011-2023

| **Variable** | **Coefficient (95% CI)** |
| --- | --- |
| **N** | 29,648 |
| **Inpatient psychiatric beds per capita** | 0.01 (95% CI: [-0.003, 0.01]) |
| **% Black** | -16.53 (95% CI: [-24.90, -8.16]; p<0.001) |
| **% Uninsured** | 40.86 (95% CI: [18.63, 63.09]; p<0.001) |
| **% Households with income under FPL** | -1.23 (95% CI: [-22.35, 19.87]) |
| **Rural** | 329.87 (95% CI: [152.67, 507.08]; p<0.001) |
| **Year** | -109.09 (95% CI: [-131.83, -86.36]) |

**Legend:**

AIC- 598404.3; BIC- 598487.2

VIFs reported in Appendix Table 2.
